# Celiac disease and obstetrical-gynecological contribution 

**Published:** 2016

**Authors:** Giovanni Casella, Guido Orfanotti, Loredana Giacomantonio, Camillo Di Bella, Valentina Crisafulli, Vincenzo Villanacci, Vittorio Baldini, Gabrio Bassotti

**Affiliations:** 1 *Medical Department, Desio Hospital ASST-Monza, Desio (Monza Brianza), Italy*; 2 *Obstetric and Gynecological Department, Desio Hospital ASST-Monza, Desio (Monza Brianza), Italy *; 3 *Pathology Department, Carate Brianza Hospital ASST Vimercate, Carate Brianza (Monza Brianza), Italy*; 4 *Institute of Pathology, “Spedali Civili” Brescia, Brescia, Italy*; 5 *Gastroenterology and Hepatology Section, Department of Medicine, University of Perugia Medical School, Perugia, Italy *

**Keywords:** Celiac disease, Infertility, Recurrent abortions

## Abstract

Celiac disease (CD) shows an increased prevalence in female, particularly during the fertile period. Celiac disease should be researched in infertility, spontaneous and recurrent abortions, delayed menarche, amenorrhea, early menopause, and children with low birth-weight. Celiac disease is still little considered during the evaluation of infertility. Up to 50% of women with untreated CD refer an experience of miscarriage or an unfavorable outcome of pregnancy. Celiac patients taking a normal diet (with gluten) have a shorter reproductive period. Women with undiagnosed CD had a higher risk of small for gestation age infants very small for gestational age infants and pre-term birth when compared with women with noted CD. The link between NCGS and infertility is actually unknown. The goal of our work is to perform an actual review about this topic and to increase the awareness in the medical population to research celiac disease in selected obstetric and gynecological disorders.

## Introduction

 Celiac disease shows an increased prevalence in females, particularly during the fertile period (1). A delayed menarche, an early menopause, higher incidence of amenorrhea, infertility and spontaneous abortions are often observed in celiac patients. Indeed, 15% of all pregnancies may lead to spontaneous abortion and the etiology may be identified in 60% of all cases (2). Abortion may be defined “recurrent” if happened two or more times. Delayed intrauterine fetal growth, characterized by a weight less than the 10th percentile, may be due to intrinsic factors as endometrial vascular abnormalities or to an altered mechanism within the endometrium of the trophoblast (2).

Celiac disease should be researched in the following clinical conditions:

1. Infertility (after ruling out anatomic alterations, endocrine disorders, infectious diseases as chlamydia trachomatis, tuberculosis, endometriosis, etc.)
2. Spontaneous and recurrent abortions (including: anti phospholipid antibody syndrome that, in some cases, may be associated with celiac disease)
3. Delayed menarche, amenorrhea and early menopause
4. Children with low birth weight
5. Miscellaneous

**Infertility**

Celiac disease (CD) is scarcely considered during the evaluation of infertility (3). In the North America 7.4-14% of women are infertile and 15% of this infertility is attributed to unexplained factors after hormonal and anatomical causes have been ruled out (4). In these cases, females are completely asymptomatic for CD and the infertility may be the only clinical expression. Many females show a mean diagnosis age of 40-50 years (3). Considering that the CD may be diagnosed with a delay up to 10 years, the entire cycle of reproductive life is lost in women with undiagnosed CD (3). The prevalence of CD in unexplained infertility may be as high as 4-8% (5,6). The Infertility in untreated CD may be related to malabsorption of Iron and/or Folate, and vitamin deficiency (7). The diagnosis of CD is often done in women without classic malabsorption symptoms (8). The “silent” presentation causes a diagnostic delay with a prolonged dietary gluten exposure that may lead to a reduction of the fertile life period (9). The First description of an association between CD and reproductive abnormalities was made by Morris, et al. in 1970 when they described three patients with untreated CD and infertility, who became pregnant after starting the gluten free diet (GFD) (10). Collin, et al. (5) studied 98 women with an unexplained infertility and they found four females affected by CD (4.1%) (p=0.02). Melloni, et al. (6), in a similar study, found two CD patients (8%) among 25 patients, who were screened for unexplained infertility. Machado, et al. (11), in a cross-sectional study of infertile women in Brazil found a 10.3% of celiac seropositivity. Tiboni, et al. (12), in an Italian study researching the prevalence of CD in women undergoing assisted reproductive techniques (ARTs), found five women with histologically diagnosed CD, but the same authors admitted that this study had not enough statistical significance due to insufficient sample size. Several studies evidenced a trend toward an increased prevalence of CD in women with infertility (17 out of 641 women studied- 2.4%) than healthy controls (20 out of 2167- 0.9%) (8). Jackson, et al. (13), in a cohort study of women with unexplained infertility in in the general U.S. population, found the presence of anti endomysial antibodies (EMA) only in one patient (0-8%). This data is similar to the prevalence of CD in the general U.S. population (13). Khoshbaten, et al. in their study in Iran, evidenced a higher frequency of CD among unexplained infertile couples compared with fertile couples (14). These data were confirmed by Shamaly, et al. (15) that, in an Arabian study about 192 women affected by unexplained infertility, found 2.65% of all these patients affected by CD with a percentage 5 times higher than the control (0.5%). It is important to underline that almost all these CD patients did not refer gastro-intestinal symptoms. Although GFD in these patients might resolve the obstetric-gynecological problems related to CD, this does not happen in all patients. The potential of a GFD to have a positive effect on fertility is rationalized by the improvement of nutritional imbalance that includes malabsorption and blood deficiency of zinc, selenium, iron, folate; these elements may be important to develop celiac disease-mediated reproductive disorders (16). In many patients, diagnosis of CD may be done during or after the pregnancy; 11% of celiac patients show the first symptom of the disease during the pregnancy (1). In the past, celiac females not following GFD were considered as “Infertile”, while Ferguson and Coll (7) have demonstrated that the percentage of pregnancies in this population is similar at general population. 

**Spontaneous and Recurrent Abortion**

 Up to 50% of women with untreated CD refer an experience of miscarriage or an unfavorable outcome of pregnancy (2). Sher, et al. (17) noted an increased incidence of spontaneous abortions. In adult patients with CD, the disease has insidious clinical manifestations with few symptoms and the pregnancy may be considered a “trigger” to unmask the disease (5), that it may be considered as “latent”. Zinc deficiency may determine impaired synthesis and secretion of luteinizing hormone (LH) and follicle- stimulating hormone (FSH) that leads to the abnormal ovarian axis, secondary amenorrhea, spontaneous abortion, and pre-eclampsia (18). Selenium deficiency affects the synthesis and secretion of FSH and LH (18). Folic acid is an essential vitamin in nucleic acid metabolism and its deficiency has a negative impact on rapidly proliferating tissues in the embryo, especially in neuronal cell development (19). The severity of malnutrition correlates directly with the frequency of gynecologic and obstetric disorders. Although women with infertility and CD with histologically total and sub-total villous atrophy do not often display clinical signs of malnutrition and/or micronutrient deficiency (15). CD patients on free-diet generally show increased blood levels of autoantibodies, in particular anti transglutaminase (anti TtG) antibodies (20). Anti TtG could be directly involved in placental-related pregnancy complication (19). Enzyme TtG is expressed in many different tissues and organs such as intracellular or extracellular (19). TtG is also expressed in endometrial cells, stromal and trophoblast placental cells with higher levels in late pregnancy (21). TtG is involved in extracellular matrix assembly and cell adhesion, spreading and migration in diverse tissues (22). TtG localized on syncytiotrophoblast may be a target of maternal autoantibodies in CD. The binding of circulating anti TtG antibodies to placenta cells could be an immunologic mechanism that may interfere with pregnancy outcome in CD patients (19). Normal development and function of the placenta requires the invasion of maternal decidua by extravillous trophoblasts (EVT) followed by abundant and organized vascular growth (23). EVTs produce a large amount of basic proteins and hormones involved in the maintenance of pregnancy (19). It is likely that increased apoptosis of EVTs may contribute to the pathophysiology of human miscarriage and intra uterine growth retardation (IUGR) (24) in celiac women on free-diet due to an increased apoptosis of EVT in placenta with a subsequent low birth weight of newborns. Anti TtG antibodies may cause an inhibition of syncytial TtG, with impairment of placental development (19). Endometrial angiogenesis and decidualization are important steps for a successful implantation and a good outcome of pregnancy. The presence of clinical signs during the pregnancy and puerperium, such as iron deficiency anemia (IDA), episodes of diarrhea and oral ulcerations should advise the physician to consider the presence of the CD. Molteni, et al. (25) noted an increased percentage of recurrent spontaneous abortions in untreated celiac patients, not correlated to clinical severity and biochemical alterations of the pathology. To date, Screening for CD is not contemplated in pregnant women. Martinelli, et al. (26) reported a higher prevalence of CD compare to the past (1.42% against 0.4-1%); in this study conducted in 845 pregnant women, the authors found 12 celiac patients and 7 of these referred an unfavorable pregnancy outcome; 4 of 5 multiparous undiagnosed women referred a prior miscarriage (26). Moleski, et al. (9) noted that most (85%) of the spontaneous abortions in CD women occur before starting the GFD. Ciacci, et al. (27) demonstrated that women with undiagnosed CD have an 8.9 relative risk of abortion compared with treated patients. Gasbarrini, et al. (2) reported that 8% (3 out of 40 studied patients) of the CD prevalence in females with recurrent spontaneous abortions, defined as the presence of 2 or more consecutive spontaneous abortions of unknown origin. Antiphospholipid antibodies, the most commonly detected of which are lupus anticoagulant, anti cardiolipin and anti-beta 2 glycoprotein 1 antibodies, are associated with the so-called “antiphospholipid syndrome” clinically characterized by arterial and venous thrombotic disease, thrombocytopenia and fetal wastage (28). Antiphospholipid antibodies are found in young healthy subjects with a prevalence of 1-5%, which may increase with age and is higher in patients with autoimmune diseases (29). Untreated CD patients may have an increased prevalence of anticardiolipin antibodies (14%) (30). Antiphospholipid Syndrome is associated with an unusually high proportion of pregnancy loss after the 10th week of gestation (31). The association between “antiphospholipid syndrome” and CD has been reported only in the case report (31) and larger studies are needed to establish the exact prevalence of this syndrome in CD patients. 

**Delayed menarca, amenorrhea and early menopause**

Celiac patients on a normal diet have a shorter reproductive period than the control population (17). The age of menarche in celiac girls has been reported to be delayed for more than 2 years as noted in the case-control study conducted by Collin, et al. (5). Sher and Mayberry investigated the age at menarche and menopause. They also noted that the mean age at menarche of CD patients was significantly higher (13.6 years vs 12.7 years in controls) and the mean age at menopause in CD patients and controls was 47.6 and 50.1 years, respectively (17). Molteni, et al. (25) investigated 54 women with untreated CD compared to 54 healthy controls, finding that the mean age of menarche was delayed in untreated patients with respect to controls (13.5 years vs 12.1 yeras); 38.8% of these patients referred amenorrhea compared with 9.2% of controls (25). Again, in Molteni’s study (25) the mean age of menopause onset was lower in patients with untreated CD (45.5 years) than in controls (49.5 years), but the difference was not statistically significant probably related to the small number of studied subjects (25). Ferguson, et al. (7), in their study on 74 celiac women, noted that 20 patients on GFD had a lower age at menarche (13.5 +- 1 SD; p=0.01) compared to 54 women with CD but on free diet (15± 2 SD).Twenty-two of these patients reached menopause: 6 on GFD and 16 on a normal diet; celiac patients on GFD showed a significantly later menopause (53± 1.2 SD vs 45± 5.5 DS; p=0.0001). In Ferguson’s study Amenorrhea, unrelated to pregnancy and of over 3 months duration, was present in 16 of 54 celiac patients on normal diet and only in 2 patients on GFD (7). Secondary amenorrhea was found in CD patients independently of the degree of malnutrition and Molteni, et al. found that the percentage of the CD was higher in well-nourished patients (38.8%) (25). In a recent study, 24 % of celiac women reported a past history of at least one menstrual cycle disorder vs 10 % of controls (p=0.038), with a higher percentage of unexplained dysfunctional uterine bleeding present in celiac women; more than 80% of these patients stopped uterine bleeding after gluten free diet (32). CD patients may be eutrophic or have mild to severe malnutrition related to multiple factors as site of the lesions, length of the intestine involved in the disease, grade of malabsorption and interval between the appearance of first symptoms and the correct diagnosis (33). Functional hypopituitarism and atrophy of reproductive organs are associated with malnutrition and weight loss that may determine an impaired concentration of gonadotrophins and estrogens (34). When CD patients are compared with patients with different malabsorption syndromes, the endocrine abnormalities are more evident in CD patients without malnutrition suggesting that circulating gluten peptides may contribute to reduce the hypothalamic pituitary regulation of gonadal function (35) independently of the general nutritional status (36). Gluten ingestion plays a central role in modifying the immunologic response on an individual basis without relation to the age of the diagnosis or to the duration of contact with gluten (37). 

**Pre-Term Delivery and Low Weight Children at Birth**

Moleski, et al. demonstrated that CD women have a higher prevalence of pre-term deliveries (9). Ludvigsson, et al. (38) found that not diagnosing CD before the start of pregnancy may be associated with an increased risk of low birth weight (< 2500 g), very low birth weight (<1500 g) and pre-term birth. An Italian study (39) on 868 women with pre-term or low birth weight babies found that 1.6% of all these women had an undiagnosed CD with an incidence greater 2.25 times than the control population (p <0.04) (22). It is important to underline that less than 20% of women with undiagnosed CD showed gastrointestinal symptoms (39). A large Danish population-based cohort study screened 504.324 babies including 346 born to women with diagnosed CD and 1105 born to women with undiagnosed CD (40). Women with undiagnosed CD had a higher risk of small for gestation age infants (OR: 1.31; 95% CI: 1.06-1.63), very small for gestational age infants (OR: 1.54; 95% CI: 1.17-2.03) and pre-term birth (OR: 1.33; 95% CI: 1.02-1.72) when compared with women with known CD (37). Infants with undiagnosed CD had a reduced mean birth weight of 100 g (adjusted difference: 98; 95% CI: -130 to – 67). Norgard, et al. (41), in pregnant women with CD, noted an incidence of small for gestational age (SGA) neonates of 6-8% in celiac patients and 2-3% in the controls (OR: 1.6-3.4). Gasbarrini, et al. (2) observed a prevalence of CD about 15% in women with SGA neonates. Ciacci, et al. (27) have demonstrated the utility of GFD on pregnancy outcome through a comparison between the 31 CD treated women versus 94 CD untreated women with a higher rate of low-birth-weight babies (RR: 5.84; 90% CI: 1.07-31.9) and this percentage was unrelated to the severity of the CD so clinically as histologically (27). In the same study, a secondary analysis about 12 pregnant women with CD showed a reduced number of low birth-weight babies from 29.4 to 0% (p< 0.05) (27). It is important to underline that in many of these studies the patients did not show classic symptoms of CD, confirming the importance of a serological screening to identify CD patients (26). Norgard, et al. (41) reported that serological screening for CD in women with intrauterine growth retardation of unknown etiology may also determine a better outcome of pregnancy. Greco, et al. (42), instead, found a high rate of undiagnosed CD in pregnant women without excess risk of abortion, premature delivery, small birth weight or intrauterine growth retardation. Dhalwani, et al. (43) studied 2,426,225 women during their fertile period and did not find differences between treated and untreated CD patients regarding fertility problems, except when the diagnosis of CD was made between 25-39 years of age. Wolf, et al., in their study on 103 pregnant women studied for SGA neonates, found only 1 patient affected by CD, and concluded that the relative risk of CD in SGA neonates is low (44). Considering that the data on the effects of dietary treatment are conflicting, the authors suggested that serological screening for sub-clinical CD before or at the beginning of pregnancy is not indicated. Similarly, the screening test is not routinely indicated in women after delivery of SGA neonates. Marild, et al. (45) found a 21% increased risk of CD in children with SGA due to multi-factorial conditions. Steinborn, et al. (46) described an altered cell mediated immunological development that may influence the intestinal immunity. 

**Miscellaneous**

Wolf et al., in a study investigating 131 women admitted for severe pre-eclampsia and delivery before 34 weeks of gestational age, found only 1 woman affected by CD (45), and suggested that serological screening for CD is not routinely indicated in pregnant women after an episode of Pre-Eclampsia. Sultan et al (47) found a 34% increased risk of post-partum hemorrhage and assisted delivery among pregnant women diagnosed with CD. However, these authors reported no increased risk of cesarean section in the undiagnosed CD group in contrast to the data reported by Lundgvisson et al (38) where an increased risk of Cesarean section was present, probably related to the difference of medical indications between United Kingdom and Sweden. Mothers with CD have an increased cesarean delivery rate (39). 

**Non Celiac Gluten Sensitivity (NCGS) and Reproductive Disorders**

Non celiac gluten sensitivity (NCGS) is defined by clinical evidence of improvement of symptoms following the introduction of gluten free diet (GFD) in the absence of Enteropathy (48). Autoantibodies as TG2 are absent in NCGS. Anti gliadin antibodies (AGA) may be an indicator of NCGS in more than 50% of all patients presenting to the gastroenterologist, particularly IgG AGA (49). The link between NCGS and Infertility is actually unknown (50). It is possible that the diet may influence reproductive immunology. Bold and Rostami (50) reported the case of a couple who had the possibility to conceive a son after 1 year of being on a gluten free diet and six tentative of assisted reproductive technique (ART) (50). Further researches are needed to study the role and the relative pathophysiological mechanisms of NCGS in infertility in men and women (50). Patients of childbearing age with fertility problems should be informed that a GFD might improve their fertility (50). 

**Table 1:** When celiac disease should be investigated in obstetrical-gynecological disorders?

Clinical condition	References
Infertility	5, 6, 11, 12
Spontaneous and Recurrent Abortion	2, 25, 26, 27
Delayed menarca	5, 13, 17
Amenorrhea	7
Early menopause	13, 17, 25
Pre term deliveries	9
Low birth weight	38, 39,40,41
Iron deficiency anaemia unresponsive to oral iron and more accentuated in second pregnancy	14
Dysfunctional rectal bleeding	32

## Discussion

The presence of clinical signs, during the pregnancy and puerperium, as iron deficiency anaemia (IDA), some episodes of diarrhea and oral ulcerations should advise the Physician to consider the presence of CD. Ferguson and colleagues (7) reported that some patients, during the pregnancy, may show a partial regression of CD. On the other hand, Pauznere et al., (33) described two patients with appearance of symptoms 6-8 weeks after the delivery, probably due to a possible relapse of autoimmune disorders as systemic lupus erythematous caused by a maternal contact with fetal antigens or a modified hormonal status (5). During the pregnancy, CD may cause an increased blood deficit of Folate, Vitamin B12 and a following impairment of nutritional condition (7). However, most pregnant women do not show clinical signs of Malnutrition (8). Gasbarrini, et al. (2) reported that 15% of intrauterine delayed fetal growth and 4% of all infertile females without a known etiology may be affected by CD (8). In conclusion, increase the awareness in the medical population to research on celiac disease in selected obstetric and gynecological disorders is recommended.
